# A Computational Model of the Fetal Circulation to Quantify Blood Redistribution in Intrauterine Growth Restriction

**DOI:** 10.1371/journal.pcbi.1003667

**Published:** 2014-06-12

**Authors:** Patricia Garcia-Canadilla, Paula A. Rudenick, Fatima Crispi, Monica Cruz-Lemini, Georgina Palau, Oscar Camara, Eduard Gratacos, Bart H. Bijens

**Affiliations:** 1 BCNatal - Barcelona Center for Maternal-Fetal and Neonatal Medicine (Hospital Clínic and Hospital Sant Joan de Déu), IDIBAPS, University of Barcelona, and Centre for Biomedical Research on Rare Diseases (CIBER-ER), Barcelona, Spain; 2 Physense, DTIC, Universitat Pompeu Fabra, Barcelona, Spain; 3 University Hospital and Research Institute Vall d'Hebron, Universitat Autònoma de Barcelona, Barcelona, Spain; 4 ICREA, Barcelona, Spain; Tel Aviv University, Israel

## Abstract

Intrauterine growth restriction (IUGR) due to placental insufficiency is associated with blood flow redistribution in order to maintain delivery of oxygenated blood to the brain. Given that, in the fetus the aortic isthmus (AoI) is a key arterial connection between the cerebral and placental circulations, quantifying AoI blood flow has been proposed to assess this brain sparing effect in clinical practice. While numerous clinical studies have studied this parameter, fundamental understanding of its determinant factors and its quantitative relation with other aspects of haemodynamic remodeling has been limited. Computational models of the cardiovascular circulation have been proposed for exactly this purpose since they allow both for studying the contributions from isolated parameters as well as estimating properties that cannot be directly assessed from clinical measurements. Therefore, a computational model of the fetal circulation was developed, including the key elements related to fetal blood redistribution and using measured cardiac outflow profiles to allow personalization. The model was first calibrated using patient-specific Doppler data from a healthy fetus. Next, in order to understand the contributions of the main parameters determining blood redistribution, AoI and middle cerebral artery (MCA) flow changes were studied by variation of cerebral and peripheral-placental resistances. Finally, to study how this affects an individual fetus, the model was fitted to three IUGR cases with different degrees of severity. In conclusion, the proposed computational model provides a good approximation to assess blood flow changes in the fetal circulation. The results support that while MCA flow is mainly determined by a fall in brain resistance, the AoI is influenced by a balance between increased peripheral-placental and decreased cerebral resistances. Personalizing the model allows for quantifying the balance between cerebral and peripheral-placental remodeling, thus providing potentially novel information to aid clinical follow up.

## Author Summary

Intrauterine growth restriction (IUGR) is one of the leading causes of perinatal mortality and can be defined as a low birth weight together with signs of chronic hypoxia or malnutrition. It is mostly due to placental insufficiency resulting in a chronic restriction of oxygen and nutrients to the fetus. IUGR leads to cardiac dysfunction in utero which can persist postnatally. Under these altered conditions, IUGR fetuses redistribute their blood in order to maintain delivery of oxygenated blood to the brain, known as brain sparing. Given that, in the fetus the aortic isthmus (AoI) is a key arterial connection between the cerebral and placental circulations, quantifying AoI blood flow has been proposed to assess this brain sparing effect in clinical practice. However, which remodeling or redistribution processes in the cardiovascular systems induce the observed changes in AoI flow in IUGR fetuses is not fully understood. We developed a computational model of the fetal circulation, including the key elements related to fetal blood redistribution. Using measured cardiac outflow profiles to allow personalization, we can recreate and better understand the blood flow changes in individual IUGR fetuses.

## Introduction

Intrauterine growth restriction (IUGR), predominately due to placental insufficiency, is one of the main causes of perinatal mortality and morbidity [1], [2], and defined as a birth weight below the 10th percentile for gestational age. IUGR fetuses, suffering from hypoxia and undernutrition, show Doppler changes in several arteries of the feto-placental circulation such as umbilical artery (UA), middle cerebral artery (MCA), and also in the aortic isthmus (AoI). These changes are assessed in clinical practice to stage the severity of IUGR and are thought to reflect blood flow redistribution due to increased peripheral resistance, with decreased brain resistance in order to maximize brain blood supply under an adverse environment. In IUGR fetuses, AoI diastolic forward flow usually decreases and can become reversed in the more severe cases reflecting blood redistribution from the ductus arteriosus towards the brain instead of the periphery. Reverse flow in the AoI is associated with worse perinatal and neurodevelopmental outcome [3]–[7]. However, which remodeling or redistribution processes in the cardiovascular system induce the observed changes in AoI flow in IUGR fetuses is not fully understood. It has been proposed that cerebral vasodilation plays a major role in decreasing diastolic flow in the AoI [8]. However, other clinical studies suggested that AoI flow pattern is influenced by simultaneous changes in both cerebral and peripheral-placental resistances [6], [9]–[11]. Moreover, some experimental studies in an ovine animal model [12]–[14] have quantified the influence of placental resistance increase in the AoI flow, showing a strong correlation between the increase in the placental resistance and the amount of diastolic reversal flow in the AoI. However, the cerebral vasodilation occurring as response to fetal hypoxemia also influences the flow patterns in the AoI, and this influence could not be isolated from those changes caused by the increase in placental resistance.

Therefore, it would be of interest to be able to estimate the separate influence of the individual contributors, such as the cerebral vasodilation and placental resistance increase, on the AoI flow. This can further improve the understanding of the flow changes in IUGR and provide more targeted assessment of flow redistribution. For this, lumped computational models have been proposed to recreate and better understand hemodynamic changes in the fetal circulation [15]–[22]. These models are based on the idea that the flow in a tube is analogous to the current in an electrical circuit and flow properties such as viscosity, inertia and compliance can be modeled with resistors, inductors and capacitors respectively. Hence, the different parts of the fetal circulation, such as arteries or vascular beds, can be modeled with a set of electrical components. The parameters of the electrical components are calculated based on the cardiovascular system's physical properties and dimensions together with physiological and imaging measurements, where possible. Thus, an equivalent electric circuit of the fetal circulation can be obtained. Computational models have the advantage that they enable to evaluate the effects of changes in individual parameters on the total system performance. For example, the influence of changes in placental and/or brain resistance on the AoI flow can be evaluated separately, which cannot be performed in a clinical or experimental setting.

Previously published models of the fetal circulation focused either on the materno-fetal circulation, studying oxygen exchange [15], [17], [18]; only focused on the fetal circulation under normal conditions [16], [20]; lack the full complexity of the fetal circulation (such as the ductus arteriosus) to study the flow redistribution in the places connecting the specific segments present [18], [21]; or have used a simplified and non-measured and personalized flow waveform at the entrance of the aorta and pulmonary artery [19], [21], [22].

Therefore, we developed an extended lumped model of the fetal circulation, including all relevant components to study flow redistribution in IUGR that additionally can be tuned towards an individual fetus when blood flow measurements are available. This model was further used to help in better understanding the hemodynamic changes induced by altered conditions in the brain circulation, including the AoI and the MCA. The model was calibrated and validated using clinical measurements from a healthy control fetus. Next a parametric study was performed to specifically evaluate contributors to flow changes in the AoI and cerebral arteries (CA). Finally, the model was personalized with clinical data from three IUGR cases with different degrees of severity in order to show the individual contributors to flow redistribution.

## Materials and Methods

### Study individuals

In order to calibrate and validate the accuracy of the fetal circulation model, and to show its potential for personalization, clinical and Doppler data from one control and three IUGR fetuses with different degrees of severity were included. Eligible cases were singleton pregnancies that were selected from women who attended the Maternal-Fetal Medicine Department at Hospital Clínic de Barcelona. The study protocol was approved by the local Ethics Committee and patients provided written informed consent. IUGR was defined as an estimated fetal weight [23] and confirmed birth weight below the 10^th^ percentile according to local reference curves [24] together with a pulsatility index (PI) in the UA above 2 standard deviations [25]. IUGR fetuses were classified in stages of severity based upon the end-diastolic flow (EDF) in the UA as: present (PEDF), absent (AEDF) or reversed (REDF) [26]. We selected one IUGR representative of each severity stage.

All IUGR cases and the control fetus underwent an ultrasonographic examination between 31–34 weeks of gestation using a Siemens Sonoline Antares machine (Siemens Medical Systems, Malvern, PA, USA) which included estimation of fetal weight, standard obstetric Doppler evaluation and fetal echocardiography. Fetal echocardiography included the evaluation of flow velocities in the UA, MCA, AoI, ductus arteriosus and ascending aorta (only in the control fetus). The UA was evaluated in a free loop of the umbilical cord. The MCA was measured in a transverse view of the fetal skull at the level of its origin from the circle of Willis [27]. The cerebroplacental ratio was calculated by dividing MCA and UA PI [25]. PI was calculated as: systolic velocity minus diastolic velocity divided by time-averaged maximum velocity. Ductus arteriosus and AoI flow velocities were obtained either in a sagittal view of the fetal thorax with a clear visualization of the aortic arch or in a cross section of the fetal thorax at the level of the 3-vessel and trachea view [28]. The AoI flow velocity was quantified by measuring the AoI PI and flow index (IFI). The IFI was calculated as: (systolic + diastolic)/systolic velocity integrals. Aortic inflow velocity was imaged in an apical or basal 5-chamber view of the heart, and pulmonary artery inflow velocity was obtained in a right ventricular outflow tract view. Peak systolic velocities of both aortic and pulmonary artery inflows, ejection time and heart rate were measured. The diameters of the aortic and pulmonary valves were measured in frozen real-time images during systole by the leading-edge-to-edge method [29]. Additionally, Doppler recording from the ascending aorta was obtained in the control fetus. The angle of insonation was kept as close as possible to 0° and always below 30°. Doppler data are shown as crude values and z-scores by gestational age according to previously published normal values [25], [27], [28], [30]. Upon delivery, gestational age, birth weight, birth weight centile, mode of delivery, Apgar scores, presence of preeclampsia and length of stay at the neonatal intensive care unit were recorded.

### Lumped model of the fetal circulation

#### Building blocks

**Figure 1:**
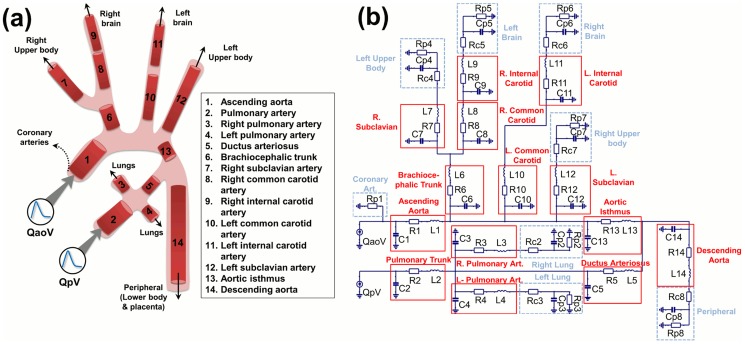
Anatomical simplified configuration and equivalent lumped model of the fetal circulation. (a) Anatomical configuration composed of 14 arterial segments and 8 vascular beds: (1) right and (2) left upper body, (3) right and (4) left brain, (5) right and (6) left lung, (7) peripheral and (8) coronary arteries. QaoV and QpV represent the aortic (left) and pulmonary (right) inflows respectively. (b) Electric circuit of the lumped model. The 14 blocks corresponding to the 14 arterial segments are highlighted in solid lines and include 1 resistor (R), 1 capacitor (C) and 1 inductor (L). The 8 blocks of vascular beds are highlighted in dashed lines and they consist of a resistor R_c_ in series with a capacitor C_p_ in parallel with a resistor R_p_.

The equivalent electrical lumped model of the fetal circulation was constructed using two main building blocks (Fig. 1B): arterial segments and peripheral vascular beds. The block describing the arterial segments included the local resistance of blood due to blood viscosity, modeled with a resistor (R), the arterial compliance modeled with a capacitor (C) and the blood inertia modeled with an inductor (L), as previously described in other cardiovascular models [21], [31], [32]. The component parameters (R, L, C) of each of the blocks modeling the different arterial segments were calculated according to their physical dimensions and properties, using the following equations: 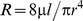, 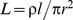 and 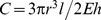, where *l* and *r* are the length and the radius of the arterial segment, *μ* is the blood viscosity calculated as *μ* = (1.15+0.075*GA)/100 [21] where GA is the gestational age in weeks, *ρ* the blood density (1.05 g·cm^−3^), *h* the wall thickness, assumed to be 15% of the arterial radius (*r*) [21], and *E* the Young's Modulus estimated from Myers and Capper [19] for the different arterial segments.

The block describing peripheral vascular beds consisted of a three-element Windkessel model. Its electrical analog circuit includes a resistor in series with a capacitor and a resistor in parallel. In the model, the series resistor R_c_ is chosen to equal the characteristic impedance of the feeding artery at high frequencies to avoid reflections at high frequencies. The parallel resistor R_p_ accounts for the organ peripheral resistance and the parallel capacitor C_p_ for the organ compliance. Additionally, a unique resistor was chosen to model the flow from the ascending aorta into the coronary arteries.

#### Anatomical configuration

The simplified fetal circulation was modeled as a set of 14 arterial segments, including the ductus arteriosus connecting the left and right circulation in the fetus, and 8 vascular beds, as shown in Fig. 1A. The peripheral vascular bed represents the combination of the fetal lower body and the placenta. Our preliminary model [22] only included 6 arterial segments and 3 simplified vascular beds, considering a unique upper body artery, but it was too limited to study the cerebral flow changes and for this reason the main upper body and cerebral arteries were included in the current model. The physical dimensions and properties of the different arterial segments were extracted from the literature [19], [33]–[44] and are listed in Table 1. The AoI was considered to be 20% of the total length of the ascending aorta and aortic arch, with a diameter equal to the distal aortic arch. Ductus arteriosus diameter was considered 1.12 times larger than AoI diameter according to [35]. Right and Left pulmonary artery length were considered 1.4 times smaller than main pulmonary artery according to [34]. Finally, according to [33] the brachiocephalic trunk diameter was considered 1.46 larger than the subclavian arteries, the right and left subclavian arteries were equals, right common carotid artery was 1.25 times larger than the left common carotid artery, the internal carotid artery length was equal to the common carotid artery length, and the its diameter was 1.17 times smaller than the common carotid artery diameter.

**Table 1:** Equations describing vessel's length and diameter as a function of gestational age (GA) in weeks, and Young's modulus (*E*) parameter for the 14 arterial segments included in the model.

Vessel	Length (mm)	Diameter (mm)	E (dyn/cm^2^)
Ascending Aorta	−8.61+0.88*GA [39]	−2.10+0.27*GA [39]	7.5·10^5^
Aortic Isthmus	−2.15+0.22*GA [39]	−1.86+0.19*GA [35]	7.5·10^5^
Descending Aorta	−34.25+3.57*GA [37], [43]	−2.22+0.22*GA [37], [43]	9.0·10^5^
Ductus Arteriosus	−2.41+0.31*GA [44]	−2.09+0.21*GA [35]	13.5·10^5^
Main pulmonary artery	−5.60+0.57*GA [38]	−2.77+0.30*GA [36]	7.5·10^5^
R. pulmonary artery	−4.00+0.41*GA [34], [38]	−1.71+0.18*GA [36]	7.5·10^5^
L. pulmonary artery	−4.00+0.41*GA [34], [38]	−1.95+0.19*GA [36]	7.5·10^5^
Brachiocephalic Trunk	−1.056+0.29*GA [40]	−1.78+0.18*GA [33], [41]	7.5·10^5^
L. subclavian artery	−2.15+0.43*GA [41]	−1.22+0.12*GA [41]	9.0·10^5^
R. subclavian artery	−2.15+0.43*GA [33], [41]	−1.22+0.12*GA [33], [41]	9.0·10^5^
L. Common Carotid artery	−9.69+1.59*GA [42]	−1.52+0.14*GA [42]	9.0·10^5^
R. Common Carotid artery	−8.25+1.36*GA [33], [42]	−1.52+0.14*GA [33], [42]	9.0·10^5^
L. Internal Carotid Artery	−8.25+1.36*GA [33], [42]	−1.22+0.11*GA [33], [42]	13.5·10^5^
R. Internal Carotid Artery	−8.25+1.36*GA [33], [42]	−1.22+0.11*GA [33], [42]	13.5·10^5^

1 dyn = 1 g·cm·s^−2^. R, L denotes right and left respectively.

#### Equivalent electrical lumped model

The equivalent electrical lumped model of the fetal circulation was implemented in Simulink, MATLAB (2013b, The MathWorks Inc., Natick, MA). It consists of a total of 64 electrical components (29 resistances, 21 capacitors and 14 inductors) and 2 inputs. The overall electrical circuit is displayed in Fig. 1B.

The electrical components of the blocks modeling the arterial segments were set up with the values calculated from the equations described in the first section, taking into account the physical properties listed in Table 1 and adjusted according to the gestational age of the fetus.

Due to the difficulty of measuring organ resistances and compliances in human fetuses, we used published data from other studies to calculate the different organ peripheral resistances (R_p_) and compliances (C_p_). Specifically, R_p_ were taken from Guettouche et al. [34] and adjusted to obtain a mean arterial blood pressure (MBP) appropriate to the gestational age of the fetus calculated as: MBP(mmHg) = 0.87*GA+10.33 [45], where GA denotes the gestational age in weeks. The initial values of the organ compliances C_p_ were estimated from Pennati et al. [20] and van den Wijngaard et al. [21] and then they were adjusted. Details about the adjustment of peripheral resistances and compliances values are included in the following sections.

#### Boundary conditions

To provide the output of the heart as input boundary condition of the computational model, the measured aortic and pulmonary artery Doppler inflow velocities from the control and IUGR fetuses were used. Maximum blood velocities were delineated from spectral Doppler images using a custom program implemented in MATLAB, providing the peak blood velocity functions in aortic and pulmonary valves (V_aoV_, V_pV_). The final blood flow inputs (Q_aoV_, Q_pV_) were calculated as: Q_aoV_ = V_aoV_ * π * r^2^_aoV_ and Q_pV_ = V_pV_ * π * r^2^_pV,_ where r_aoV_ and r_pV_ are the corresponding valve radius (aoV = aortic valve, pV = pulmonary valve).

As the fetal circulation model is not closed, venous pressure was considered 0 mmHg and therefore all the peripheral resistors and capacitors were connected to ground.

### Simulations

#### Calibration of the equivalent lumped model in normal conditions

Firstly, in order to calibrate our computational model of the fetal circulation in a healthy situation, all the circuit component parameters were set up with the values calculated as indicated in the previous sections, using the data from the control fetus (gestational age and fetal weight). Accordingly, aortic and pulmonary inflows, measured with Doppler imaging of the control fetus, were used as input functions. Model-based waveforms from AoI, CA, ductus arteriosus and ascending aorta were compared to the measured ones at these locations. The PI (PI_AoI_), IFI and percentage of reversed flow in the AoI, and also the PI in CA (PI_CA_) and in descending aorta were calculated from model-based waveforms. Finally, the amount of blood flow that was distributed towards different fetal areas, including (1) the brain, (2) the upper body, (3) the lungs, (4) the lower body and placenta and (5) the coronary arteries was calculated as the percentage of combined cardiac output (CCO) and compared with data obtained from the literature [10], [46], [47]. CCO was calculated as the sum of right and left cardiac outputs.

The overall total vascular bed resistance was adjusted to obtain a mean arterial blood pressure of 40 mmHg corresponding to a healthy fetus of 33.2 weeks of gestational age. Then, each peripheral vascular bed resistances (R_p_) were adjusted so the percentage of blood flow towards each vascular bed was within its normal range of values according to the reported data [10], [46], [47].

Regarding the peripheral vascular bed compliances C_p_, the initial values obtained from the literature [20], [21] were too high and reliable waveforms could not be obtained. Therefore, C_p_ values were estimated automatically using a constrained nonlinear optimization algorithm minimizing the relative error between the computed (denoted by ∼) and measured PI_AoI_ and PI_CA_, implemented in MATLAB. An objective function *J* was therefore defined as the sum of both relative errors as: 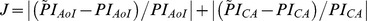. Hence, the estimation problem consisted on searching the parameters set which minimizes *J*. This process was performed iteratively until the objective function *J* was less than a predefined value. The initial parameter set was chosen randomly within a physiological range. In order to avoid local minimum solutions, we repeated the procedure several times with different initial parameter sets, and we finally chose the parameter set with a minimum value of *J*. This way, the measured Doppler waveforms from the control fetus could be reproduced.

#### Parametric study of flow changes in the AoI and CA

Next, in order to evaluate how the increase in peripheral resistance (due to placental resistance increase and peripheral vasoconstriction) and how the cerebral vasodilation influences the blood flow redistribution, peripheral resistance (Rp8 of the electrical circuit of Fig. 1B) was systematically increased up to a four-fold increase to simulate an increased placental resistance and peripheral vasoconstriction, and brain resistance (Rp5 and Rp6 of the electrical circuit of Fig. 1B) was systematically decreased up to a four-fold decrease to represent cerebral vasodilatation, keeping the remaining parameters unchanged. This range of resistance increase and decrease was chosen wide enough to cover all the possible physiological values both in normal and pathological conditions. In each simulation, IFI, PI and percentage of reversed flow in the AoI were calculated. Also, the PI in CA (PI_CA_) and in descending aorta (PI_dAo_) were calculated and the PI_dAo_/PI_CA_ ratio was obtained. Finally, the percentage of blood flow towards the brain and lower body and placenta was calculated to assess blood flow redistribution as a consequence of brain and peripheral resistance variation.

#### Patient specific modeling

Finally, the model was personalized to the three fetuses at different severity stages of IUGR. For this, the model was initialized with the specific blood inflow functions measured from each IUGR fetus. The gestational age and fetal weight of each fetus were used to compute the model parameters. Specifically, the physical dimensions of all arterial segments were calculated following the equations shown in Table 1, taking into account the gestational age of each individual. The Young's modulus of the different arterial segments was not changed. Then, in order to describe the changes of length and diameter of the fetal arterial segments as a function of fetal body weight, we scaled all the arterial dimensions according to the allometric equation: 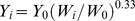 as described by Pennati et al. [48], where *W_0_* is the reference weight calculated using the relationship between fetal weight and gestational age described by Gallivan et al. [49]: *log*_10_ *(W) = *0.2508+0.1458 *GA–*0.0016*GA^2^*, where GA is the gestational age in weeks, *W_i_* is the estimated fetal weight of the IUGR fetuses (see Table 2), *Y_0_* the reference arterial dimension calculated previously, and *Y_i_* the scaled arterial dimension for the estimated fetal weight *W_i_*. Vascular bed resistances and compliances were also scaled using the allometric equations: 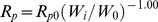 and 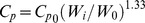 according to [48], where *R_p0_* and *C_p0_* were the vascular bed resistances and compliances calculated for the control fetus, respectively. Moreover, as *R_p0_* values were calculated to obtain a mean arterial blood pressure (MBP) of 40 mmHg, corresponding to a fetus of 33.2 weeks of gestational age, the vascular bed resistances were also scaled to obtain a MBP appropriate for each IUGR fetus.

**Table 2:** Prenatal ultrasound and perinatal characteristics of the study population.

	CONTROL	IUGR UA-PEDF	IUGR UA-AEDF	IUGR UA-REDF
***Ultrasound data***				
Gestational age at scan (weeks.days)	33.2	33.6	31.3	30.2
Estimated fetal weight (g)	2250	1500	950	500
Estimated fetal weight centile	77	<1	<1	<1
Aortic inflow peak velocity (cm·s^−1^)	91	91	62	57
Pulmonary inflow peak velocity (cm·s^−1^)	67	87	58	47
Aortic valve diameter (mm)	6.0	6.0	5.0	3.2
Pulmonary valve diameter (mm)	6.6	7.1	7.5	5.7
Heart rate (bpm)	128	159	154	144
Umbilical artery PI	0.83	1.93	2.22	3.36
Umbilical artery PI (z-score)	−0.46	3.24	4.04	7.74
Umbilical artery end-diastolic flow	Present	Present	Absent	Reversed
Middle cerebral artery PI	2.82	1.54	1.45	1.18
Middle cerebral artery (z-score)	2.36	−1.05	−1.52	−2.30
Cerebroplacental ratio	3.40	0.80	0.65	0.35
Cerebroplacental ratio (z-score)	3.24	−2.89	−4.82	−3.62
Aortic isthmus PI	3.39	4.25	6.19	27.96
Aortic isthmus PI (z-score)	1.55	3.64	9.12	69.03
Isthmus flow index	1.36	1.26	0.52	−12.36
Isthmus flow index (z-score)	0.85	0.06	−6.64	−128.27
***Perinatal outcome***				
Gestational age at delivery (weeks,days)	39.3	34.6	31.5	30.3
Birth weight (grams)	3630	1648	1060	500
Birth weight centile	87	<1	<1	<1
Gender	Female	Male	Male	Female
Fetal death	No	No	No	Yes
Cesarean section	No	No	Yes	Yes
5-minute Apgar score	10	10	8	-
Umbilical artery pH	7.28	7.28	7.20	-
Preeclampsia	No	No	No	Yes
Days in neonatal intensive care unit	0	15	10	-

IUGR, intrauterine growth restriction; UA, umbilical artery; PEDF, present end-diastolic flow; AEDF, absent end-diastolic flow; REDF, reversed end-diastolic flow; PI, pulsatility index.

All these parameters were calculated for a healthy individual. However, in IUGR fetuses, some of them can be altered, such as brain and peripheral resistances. Additionally, due to the lack of data of organ compliances in human fetuses (the values reported by Pennati et al. [20] and van den Wijngaard et al. [21] were estimated from fetal sheep circulation) we decided to include them also in the set of parameters that need to be estimated. Therefore we defined a set of 6 model parameters to estimate: (1) brain and (2) peripheral resistances, and (3) brain, (4) upper body (5) peripheral and (6) lungs compliances. To estimate these parameters we used the same constrained nonlinear optimization algorithm explained in the calibration section.

Finally, after retrieving the patient-specific parameters set, the individual IFI, PI and percentage of reversed flow in the AoI, PI_CA_, PI_dAo_/PI_CA_ and percentage of CCO towards to the brain and lower body and placenta were obtained.

## Results

### Characteristics of the study individuals

**Figure 2:**
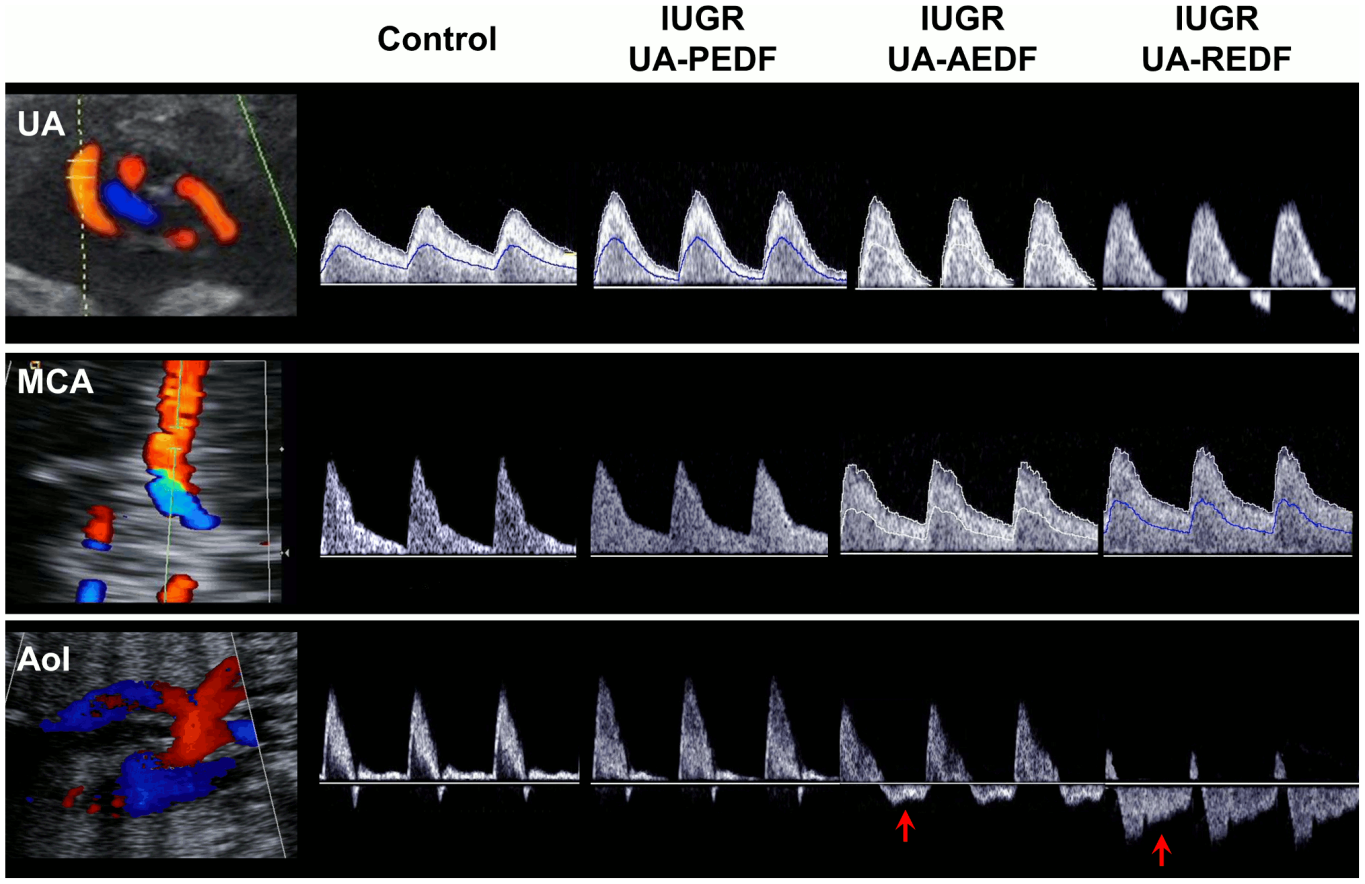
Doppler ultrasound data of the study individuals. Doppler recordings from umbilical artery (UA) (top), middle cerebral artery (MCA) (middle) and aortic isthmus (AoI) (bottom) for the control and the three intrauterine growth restricted (IUGR) fetuses with present (PEDF), absent (AEDF) or reverse (REDF) umbilical artery end-diastolic flow. Red arrows indicated reversal flow in the AoI.

Ultrasonographic and perinatal data are shown in Table 2. As expected, IUGR cases showed a higher UA PI and AoI PI, together with lower MCA PI, cerebroplacental ratio and IFI values as compared to the control fetus. Also, as expected, IUGR cases delivered earlier and had lower birthweight and birthweight centile as compared with the control fetus. There were no perinatal deaths with the exception of the most severe IUGR case (REDF) who died in utero. Fig. 2 shows the real measured flow velocity waveforms in UA, MCA and AoI for the control and the 3 IUGR fetuses. Reversed diastolic flow in the AoI can be appreciated in IUGR cases with AEDF and REDF.

### Calibration of the equivalent lumped model in normal conditions

**Figure 3:**
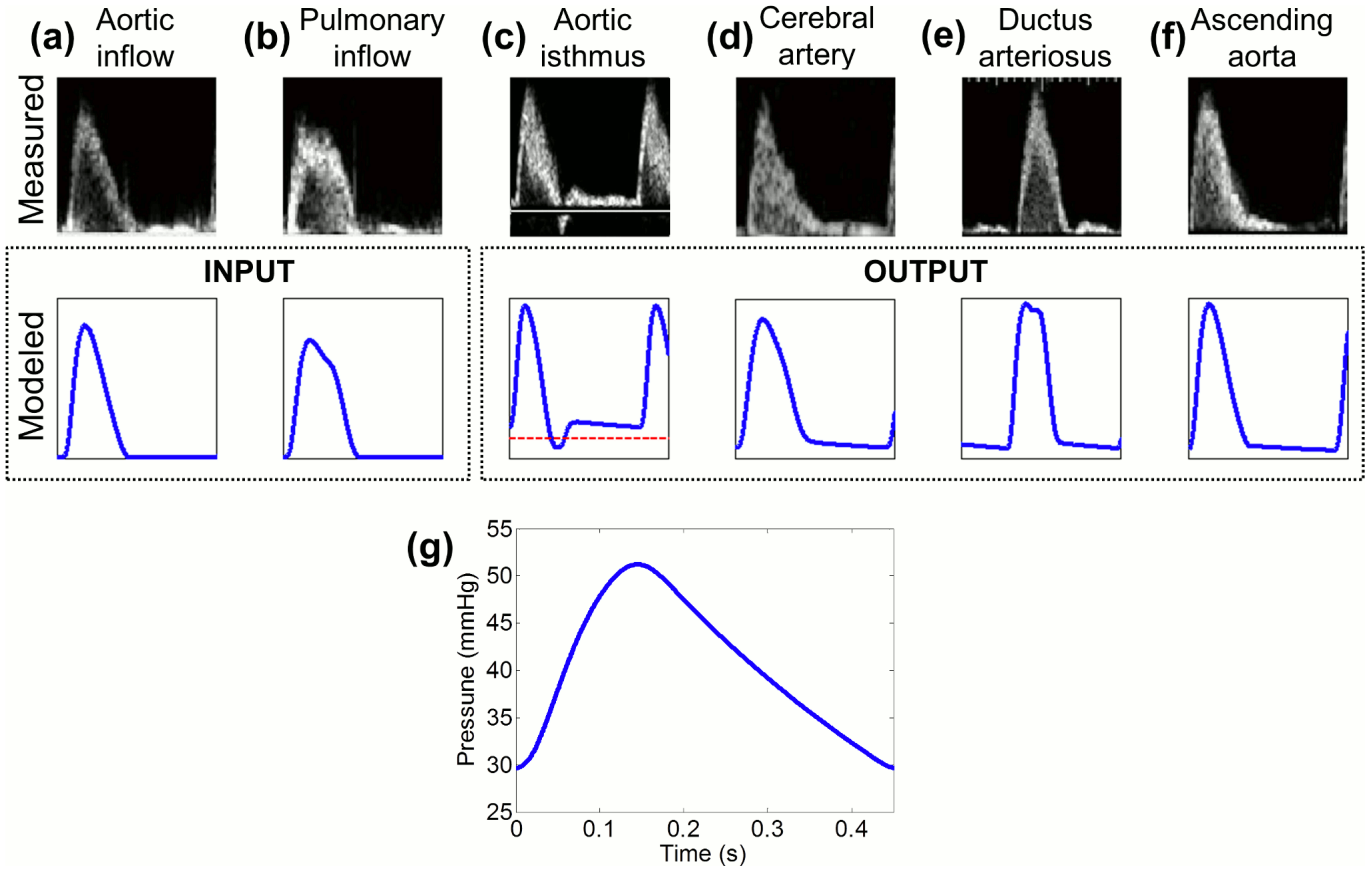
Measured and model-based flow and pressure waveforms of the control fetus. Comparison between measured and model-based blood flow waveforms in (a) aortic inflow (used as left input in our model (Q_aoV_)), (b) pulmonary inflow (used as right input in our model (Q_pV_)), (c) aortic isthmus, (d) cerebral arteries, (e) ductus arteriosus and (f) ascending aorta. Dashed line indicates a blood flow of 0 ml·s^−1^. (g) Model-based pressure waveform.

Model-based and measured flow waveforms from the aortic and pulmonary inflows, ascending aorta, AoI, MCA and ductus arteriosus of the control fetus after calibration are displayed in Fig. 3 highlighting their similarity. The model-based pressure waveform is displayed in the same figure (Fig. 3G). Furthermore, we confirmed that the amount of blood flow distributed towards each vascular bed was within the range of normal values, as shown in Table 3. The parameters values' of the vascular bed resistances and compliances are listed in Table 4.

**Table 3:** Comparison between reported and model-based combined cardiac output (CCO) distribution in a control fetus of 33 weeks of gestational age.

	Reported CCO(*)	Model-based CCO
***Anatomical segment***		
Left cardiac output	40–47%	46%
Right cardiac output	60–53%	54%
Brain	15–16%	16%
Upper body	10–14%	11%
Lungs	13–25%	15%
Lower body & placenta	50–60%	54%
Coronary arteries	3–5%	4%

*As reported in the literature [10], [46], [47].

**Table 4:** Values of the initial peripheral resistances and estimated compliances of the 8 vascular beds used to model the control and 3 IUGR fetuses.

	CONTROL	IUGR UA-PEDF	IUGR UA-AEDF	IUGR UA-REDF
**Peripheral Resistance (R_p_) (mmHg·s·ml^−1^) (initial values)**				
Brain (Rp5–Rp6)	33.87	50.90	60.39	98.71
Upper body (Rp4–Rp7)	46.44	74.20	88.04	143.89
Lungs (Rp2–Rp3)	34.52	55.14	65.43	106.93
Peripheral (Rp8)	3.75	7.49	8.89	14.54
Coronary Arteries (Rp1)	68.75	109.33	130.31	212.98
**Estimated Peripheral Compliances (C_p_) (ml·mmHg^−1^)**				
Brain (Cp5–Cp6)	0.0104	0.0052	0.0046	0.0024
Upper body (Cp4–Cp7)	0.0121	0.0083	0.0047	0.0017
Lungs (Cp2–Cp3)	0.0049	0.0038	0.0055	0.0011
Peripheral (Cp8)	0.0602	0.0242	0.0208	0.0049
**Estimated Peripheral Resistances (R_p_) (mmHg·s·ml^−1^)**				
Brain (Rp5–Rp6)	-	46.76	46.87	57.70
Peripheral (Rp8)	-	9.02	16.81	54.42

The estimated brain and peripheral resistances after the nonlinear optimization algorithm are also displayed for the IUGR fetuses.

The name of the corresponding electrical component of the circuit displayed in Fig. 1B is written in brackets.

### Parametric study of flow changes in the AoI and CA

**Figure 4:**
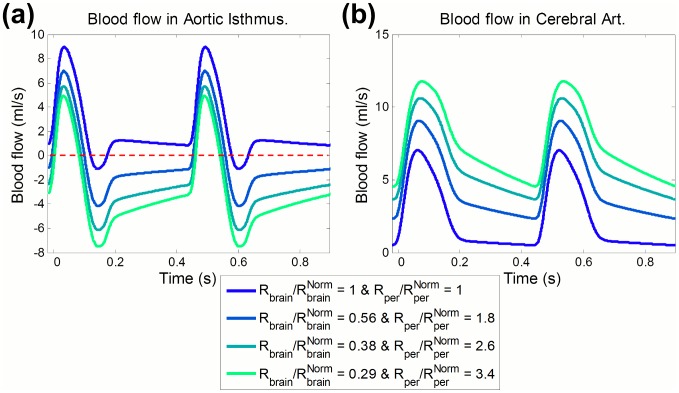
Model-based flow waveforms in the aortic isthmus and cerebral arteries. Model-based flow waveforms in the (a) aortic isthmus and (b) cerebral arteries for different degrees of peripheral and brain resistance changes. R_per_ and R_brain_ represent the peripheral and brain resistances respectively, and 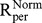 and 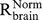 are their corresponding normal values. Dashed line indicates a blood flow of 0 ml·s^−1^.

**Figure 5:**
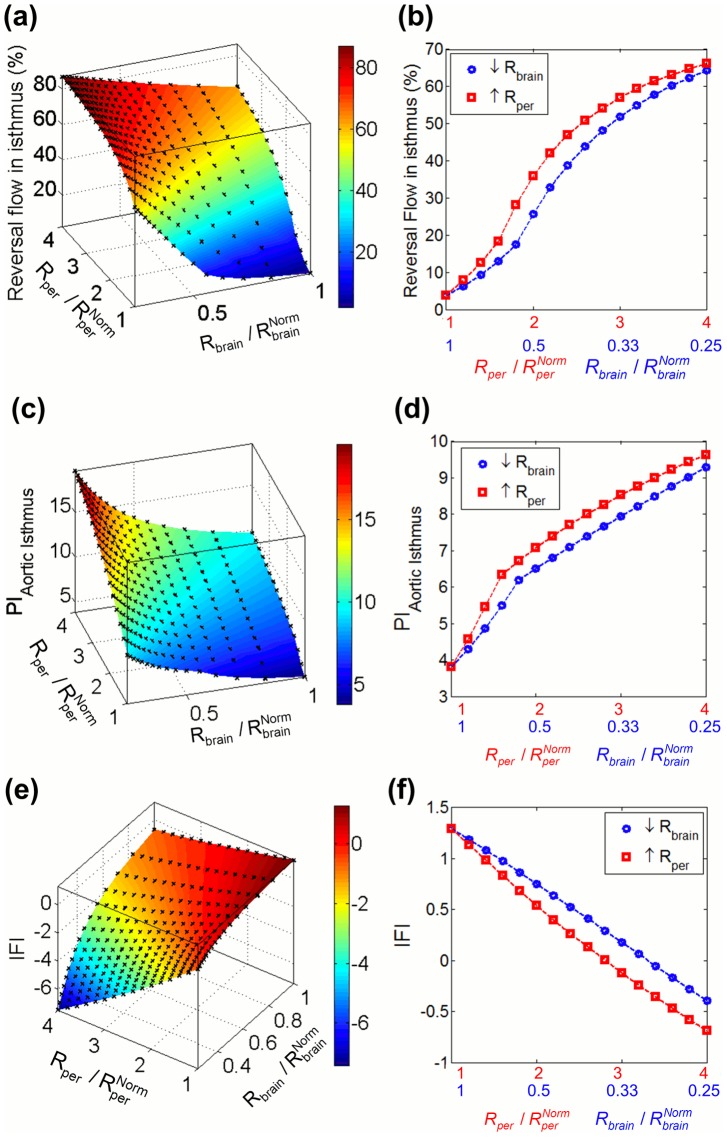
Plots of the aortic isthmus flow related indexes. Plots illustrating the percentage of reversal flow (a,b), pulsatility index (PI) (c,d) and flow index (IFI) (e,f) in aortic isthmus (AoI) as a function of decrease and increase of brain (R_brain_) and peripheral (R_per_) resistances respectively, calculated as the ratio between the current and their corresponding normal values 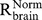 and 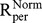. The plots in the left (a,c,e) show the variation of the three indexes as a function of all the possible combinations of R_per_ increase and R_brain_ decrease. The plots in the right (b,d,f) shows the variation of the three indexes when only one of the resistances was changed and the other was kept with a value of 1.

**Figure 6:**
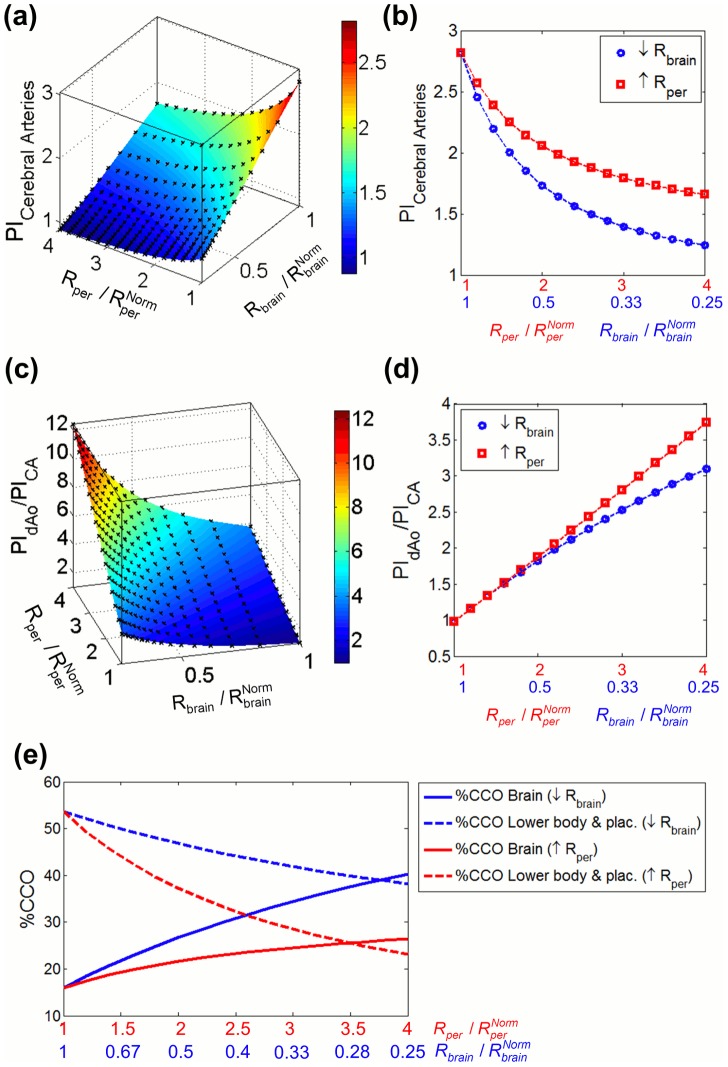
Plots of the cerebral arteries and descending aorta flow related indexes. Plots illustrating the pulsatility index (PI) in cerebral arteries (a,b) and PI ratio of descending aorta (dAo) and cerebral arteries (CA) (c,d) as a function of decrease and increase of brain (R_brain_) and peripheral (R_per_) resistances respectively, calculated as the ratio between the current and their corresponding normal values 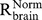 and 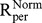. The plots in the left (a,c) show the variation of the two indexes as a function of all the possible combinations of R_per_ increase and R_brain_ decrease. The plots in the right (b,d) shows the variation of the two indexes when only one of the resistances was changed and the other was kept with a value of 1. (e) Percentage of combined cardiac output (CCO) going towards the brain (solid line) and towards the lower body & placenta (dashed line) plotted as a function of decrease and increase of brain (R_brain_) and peripheral (R_per_) resistances respectively.

Fig. 4 displays model-based AoI and CA traces for different combinations of peripheral and brain resistance values, modeling different severity degrees of IUGR. It shows that as peripheral resistance increases and/or brain resistance decreases, late-systolic and diastolic flow in the AoI is reversed while blood flow in the CA increases. The amount of reversal flow in the AoI (Fig. 5A–B), PI in the AoI (Fig. 5C–D) and IFI (Fig. 5E–F) were plotted as a function of brain and peripheral resistances relative to their normal value. Both the individual increase in peripheral resistance and decrease in brain resistance seem to have similar effects on the AoI flow. PI in the CA (PI_CA_) and the ratio between the PI in the dAo and CA (PI_dAo_/PI_CA_) were plotted in Fig. 6 as a function of brain and peripheral resistances relative to their normal value. In the case of the CA, the decrease in brain resistance seems to have a bigger influence on decreasing PI_CA_ than the increase in peripheral resistance (Fig. 6B). Regarding PI_dAo_/PI_CA,_ up to a two-fold increase/decrease in peripheral/brain resistance, its relation with the corresponding resistance variation is similar (Fig. 6D). The increase in percentage of CCO towards the brain depends mainly on the reduction of brain resistance rather than peripheral changes, as shown in the graphs plotted in Fig. 6E. However the reduction of the amount of blood flow towards the lower body and the placenta was mainly produced by the increase in the peripheral resistance.

### Patient specific modeling

**Figure 7:**
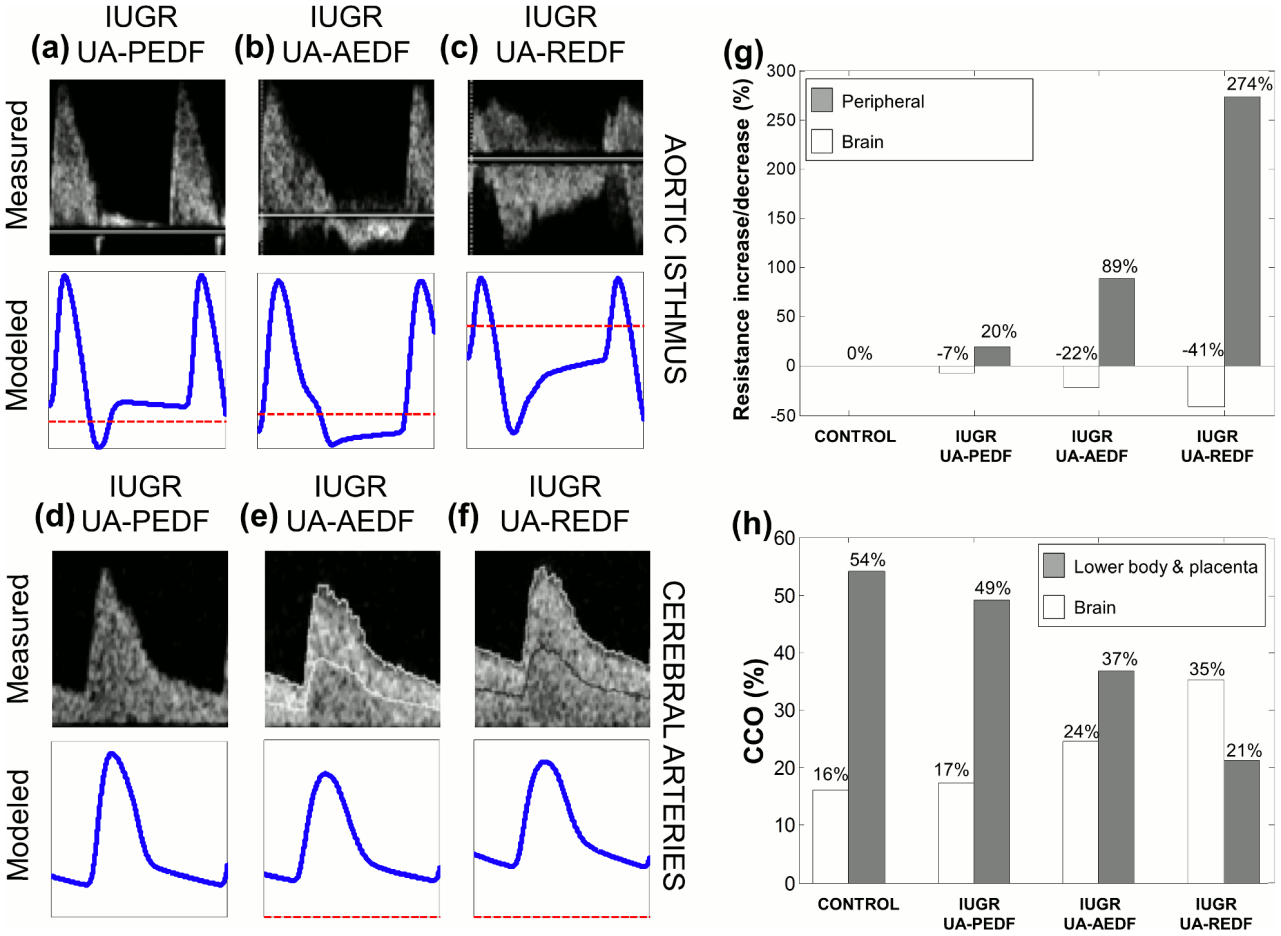
Measured and model-based flow waveforms and model parameters of the intrauterine growth restricted fetuses. Comparison between measured (top) and model-based (bottom) blood flow waveforms in the aortic isthmus (a–c) and cerebral arteries (d–f) for the intrauterine growth restricted (IUGR) fetuses with umbilical artery (UA) present (PEDF), absent (AEDF) or reversed (REDF) end-diastolic flow. Dashed line indicates a blood flow of 0 ml·s^−1^. (g) Percentage of estimated increase in peripheral-placental resistance (grey bars) and decrease in brain resistance (white bars) calculated for each individual. (h) Estimated percentage of combined cardiac output (CCO) towards the brain and towards the lower body and placenta for each individual.

The comparison between the measured and the patient specific fitting of the AoI and CA blood waveforms are displayed in Fig. 7A–F showing similar measured and model-based flow waveforms. Table 4 shows the values of vascular bed resistances and compliances and also the estimated peripheral and brain resistances after fitting for all individuals in the study. Table 5 shows the model-based parameters' values obtained for the four fetuses, which are similar to the clinical measurements shown in Table 2. The relative increase and decrease in peripheral and brain resistance respectively, estimated for each modeled case is plotted in Fig. 7G. In the IUGR fetus with UA-PEDF, the peripheral resistance was increased by 20% (×1.2) while brain resistance remained almost equal (÷1.08). In the most severe IUGR case, the peripheral resistance increased 274% (×3.7) with −41% (÷1.7) decrease of brain resistance. Therefore, in all three IUGR fetuses, the estimated variation in peripheral resistance is much higher than the variation in the brain resistance. The estimated amount of blood flow towards the brain and the lower body and placenta for the four fetuses is plotted in Fig. 7H showing that as the severity condition increases, the percentage of blood flow to the brain increases and the flow to the lower body and placenta is reduced.

**Table 5:** Modeling results for the control and the three IUGR fetuses.

	CONTROL	IUGR UA-PEDF	IUGR UA-AEDF	IUGR UA-REDF
***Model-based parameters***				
Reversal flow in aortic isthmus (%)	3%	7%	33%	91%
Isthmic flow index (IFI)	1.36	1.26	0.52	−12.35
Aortic isthmus PI	3.39	4.22	6.20	28.09
Cerebral Artery (CA) PI	2.82	1.54	1.45	1.18
Descending Aorta PI/CA PI	1.10	1.92	2.72	5.14

IUGR, intrauterine growth restriction; UA, umbilical artery; PEDF, present end-diastolic flow; AEDF, absent end-diastolic flow; REDF, reversed end-diastolic flow; PI, pulsatility index.

## Discussion

We developed a realistic computational model of the fetal circulation to study blood redistribution in IUGR enabling both parametric studies to determine individual contributors to remodeling as well as personalization to quantify changes in an individual fetus. Using this approach, we show that AoI flow changes depend both on brain vasodilation and peripheral resistance increase, while MCA flow is mainly affected by changes in cerebral resistance. Furthermore we showed that individual IUGR fetuses show marked differences in their vascular components with an exaggerated change in peripheral-placental resistance as major determinant for observed changes in measured Doppler flows.

For this, we implemented and calibrated a lumped model of the fetal circulation taking into account the main arteries of the fetal circulation, including the ductus arteriosus and the aortic isthmus. Previous approaches to model the fetal circulation focused mainly on the (oxygen) exchange within the placenta [15], [17], [18] or have only modeled fetal circulation under normal conditions [16], [20]. Some previous studies that investigated the influence of an increase in placental resistance on flow related indexes did not include the ductus arteriosus, thus limiting its comparison with in-vivo imaging and the translation to clinical practice [19], [21]. Therefore, to our knowledge, ours is the first attempt to evaluate the effect of peripheral and cerebral resistances on AoI flow by using a computational model of the fetal circulation. Another important difference from previously published models is that we use data from individual clinical imaging as boundary conditions for each modeled subject, instead of considering a single flow input with fixed values calculated from the literature. The reliability of our model was ensured by comparing the model-based flow waveforms with Doppler velocity profiles recorded in the control fetus. Moreover, the fraction of cardiac output distributed to the different peripheral regions of the fetus obtained with our model agrees with the cardiac output distribution measured in human fetuses [10], [46], [47], and also estimated in other models [16], [20].

We have comprehensively evaluated how the flow in AoI and CA are affected by changes in the vascular resistances. The increase in placenta vasculature resistance together with the vasoconstriction of the lower body arteries were modeled by increasing the peripheral resistance. The cerebral vasodilation that occurs as a compensatory mechanism to fetal hypoxia and undernutrition was modeled by decreasing brain resistance. We observed that, as increasing the modeled IUGR severity, diastolic flow in AoI decreases and, in very severe cases becomes markedly retrograde. This pattern of changes in AoI flow is consistent with the changes clinically described in IUGR cases [6], [9]–[11],[30]. We also found that the AoI flow changes observed in IUGR are influenced independently by both cerebral vasodilation and peripheral resistance increase. Previous reports suggested that the decrease in cerebral resistance plays a major role in determining the net AoI diastolic flow since MCA vasodilation precedes AoI flow abnormalization [8]. Other studies have described a correlation between the IFI and postnatal neurodevelopmental outcome [6], [7], supporting the impact of cerebral vasodilation in AoI flow changes. However, other studies [6], [9] indicated that since the AoI is connecting the two fetal circulations in parallel, AoI flow pattern reflects the existence of differences in vascular resistances, suggesting that both the increase in peripheral resistance and the cerebral vasodilation are responsible for AoI flow changes. Our results support this last hypothesis since we showed that not only cerebral vasodilation but also the increase in peripheral resistance altered the AoI flow pattern. These results are consistent with the consideration of AoI as a good predictor of not only the poor neurodevelopmental outcome [5], [6] but also of the high risk of adverse perinatal outcome and mortality [3], [7], [50].

Regarding the CA, we showed that the decrease in PI_CA_ is mostly related to cerebral vasodilation and much less influenced by changes in the periphery/placenta. This is also reflected in the amount of blood flow that is distributed towards the brain and towards lower body and placenta. For example, when brain resistance was decreased by 75%, the flow that went to the lower body and placenta was decreased by 57% but cerebral flow increased 150%, showing how cerebral vasodilation is the most responsible for the blood flow increase in CA. These results are in line with those previously reported by van den Wijngaard et al. [21] and consistent with MCA being a risk stratifying factor for suboptimal neurodevelopment in IUGR rather than perinatal complications [4], [50]. Finally, PI_dAo_/PI_CA_ showed a linear increase with the severity of IUGR, starting from a value about 1.0 for the control fetus. This result was consistent with the data published by Makikallio et al. [9] that showed also a value of 1.0 in control fetuses and an increase in the IUGR group.

Next we constructed patient-specific models of three IUGR fetuses. We were able to reproduce the AoI and CA flow waveforms and also the model-based values for AoI PI, CA PI and IFI parameters were consistent with the measured ones, demonstrating that the developed lumped model of the fetal circulation not only is able to reproduce the hemodynamic changes that occur in fetus under normal conditions, but also with increased peripheral-placental resistance and vasodilation. This is helpful to estimate parameters that cannot be measured clinically such as the relative variation of the upper and lower body resistances who might be more directly related to the staging of the disease than only the measurements of PI, currently used in clinical practice.

However, the presented lumped model has some limitations. Firstly, 0D lumped models only consider the temporal variation of pressure, flow and volume variables, assuming no variation of these parameters in the spatial dimension. However, since the aim of the study was to evaluate the hemodynamic interactions among the different cardiovascular parts, without considering flow phenomena or wave reflections, we think that 0D lumped models were accurate enough for our purpose. Also, the model is a simplified version of the fetal arterial tree because it only considers one artery and one peripheral bed for the lower body. However since the goal was to study the flow changes in the AoI and brain we considered that including or not all the lower arteries and organs would have the same effect on the AoI and cerebral flows. Secondly, the increase in right ventricular predominance observed in IUGR fetuses [10], [51], [52] was not taken into account for the parametric study as we decided to focus on the resistance variation and used the measurement of a normal fetus as input rather than changing the input for each combination of resistances. Thirdly, the fetal heart was not modeled. Nevertheless, since we used patient-specific Doppler waveforms at the ventricular outputs as the input of the model, we were indirectly considering the cardiac changes that may affect the cardiac output when studying the individual cases. Fourthly, our model does not take into account the major biochemical disorders created by placental circulatory insufficiency, such as low pH, hypoxia and respiratory acidosis, that can interfere with the normal cardio circulatory function and play a significant role in the hemodynamic changes during IUGR [53]. Fifthly, considering a venous pressure of 0 mmHg could have an effect, but mainly on the pressure values. However, we were interested only in flow waveforms and we were able to reproduce the measured Doppler traces in all cases. Finally, although we are not estimating all the parameters that may change between subjects and/or under pathological conditions, we still can consider our approach as a patient-specific modeling. We are using patient specific data to build the model and its boundary conditions (gestational age, fetal weight, heart rate, Doppler velocities and valve radius) to finally estimate the specific resistances variation for each individual.

In conclusion, the proposed equivalent lumped model seems to be a good approximation to assess hemodynamic changes in the fetal circulation under abnormal growth conditions. Further developments of the model can be useful for assessing further vessels and their interactions under various clinical conditions, and the impact of interventions. Our results suggested that AoI flow is affected by peripheral-placental as well as cerebral resistances while CA flow mainly depends on cerebral resistance. Furthermore, when personalizing the model to IUGR fetuses we were able to estimate the specific vascular resistances variation, suggesting that the peripheral-placental resistance is the major determinant for observed changes in measured Doppler flows. This study supports the potential role of AoI as marker of adverse perinatal and neurological outcome since it is a central vessel connecting the two ventricular outputs and therefore its flow reflects the balance between ventricular output and upper/lower body vascular resistances. Personalizing the model shows promise to directly assess properties of the vascular bed rather than using indirect Doppler measurements.
