# Correction: A Computational Model of the Fetal Circulation to Quantify Blood Redistribution in Intrauterine Growth Restriction

**DOI:** 10.1371/journal.pcbi.1003864

**Published:** 2014-08-28

**Authors:** The PLOS Computational Biology Staff

The eighth author's name is spelled incorrectly. The correct name is: Bart H. Bijnens.
